# Biodegradable carboxymethyl cellulose–polyvinyl alcohol composite incorporated with *Glycyrrhiza* *Glabra* L. essential oil: Physicochemical and antibacterial features

**DOI:** 10.1002/fsn3.2449

**Published:** 2021-07-05

**Authors:** Arash Bahrami, Reza Fattahi

**Affiliations:** ^1^ Department of Food Science and Technology Faculty of Agriculture University of Tabriz Tabriz Iran; ^2^ Department of Food Science and Technology Faculty of Agriculture Tarbiat Modares University Tehran Iran

**Keywords:** carboxymethyl cellulose, composite film, *Glycyrrhiza glabra* L., polyvinyl alcohol

## Abstract

*Glycyrrhiza glabra* L. root essential oil (GGEO) has well‐known antimicrobial and therapeutic features. In this study, a new antimicrobial carboxymethyl cellulose–polyvinyl alcohol (CMC‐PVA) binary film was developed using GGEO as an active compound. The effects of various concentrations of GGEO (0.25%, 0.50%, and 0.75%) were scrutinized on the physicochemical and antibacterial properties of composites. It was discovered that GGEO significantly reduced the composite ultimate tensile strength from 17.01 to 3.86 MPa. Further, by increasing the concentration of GGEO to 0.75%, the water vapor permeability and moisture content increased to 13.61 × 10^−9^ g/m s^−1^ Pa^−1^ and 41.06%, respectively. The results indicated that the active films possessed good inhibitory effects against the gram‐positive bacteria (*L. monocytogenes and Staphylococcus aureus*) and were less powerful against gram‐negative bacteria (*Escherichia coli and S. typhimurium*). Finally, the results highlighted that GGEO can act as an excellent antimicrobial agent in combination with CMC‐PVA composite.

## Graphical abstract

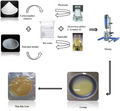

## 1 INTRODUCTION

Packaging plays an important role in protecting foodstuff against physicochemical damage and microbial activity, while also extending the shelf life (Alizadeh‐Sani et al., 2018). Generally, petrochemical compounds are the main material for food packaging. These polymers possess good mechanical properties, are well immune to oxygen and moisture, and are inexpensive. However, their use has raised concerns about environmental pollution (Otoni, Moura, et al., 2014).

As a possible promising solution, biodegradable polymeric materials can be a sound alternative for food packaging (Cazón et al., 2018). Most recent research on eco‐friendly polymeric compositions for food packaging has focused on biopolymers such as proteins, polysaccharides, and their derivatives (Ahmad et al., 2012; Dashipour et al., 2015). Recent studies have been explored the potential of carboxymethyl cellulose (CMC) films for the preparation of active packaging of foods (Fattahi & Seyedain–Ardabili, 2021; Riaz et al., 2020; Simsek et al., 2020; Wang et al., 2020).

Carboxymethyl cellulose is the most common polysaccharide used for film blending primarily because it has low‐cost and is nontoxic as well as nonallergenic (Rodsamran & Sothornvit, 2018a). CMC is acquired from alkali cellulose by etherification in the presence of monochloroacetic acid (Rodsamran & Sothornvit, 2020). It has been identified as a GRAS anionic biopolymer (E466) which is used extensively in the food and pharmaceutical industry for applications such as filming, emulsification, suspending, thickening, and stabilizing (Rodsamran & Sothornvit, 2018b). Several studies have revealed superior film‐forming properties of CMC (El‐Sayed et al., 2011; Muppalla et al., 2014; Raeisi et al., 2015). Nevertheless, the films formed by it have poor mechanical and barrier properties. The combination of decomposable polymers with different properties and structures seems to be an appropriate solution to overcoming these shortcomings (Cazón et al., 2018; Fattahi et al., 2020).

Polyvinyl alcohol (PVA) is one of the semi‐crystalline synthetic polymers whose backbone is composed predominantly of carbon chains and is thus completely biodegradable. It is a nontoxic polymer with a zigzag structure. Various studies have shown its good chemical and mechanical stability, transparency, thermal stability in a wide range of temperatures, processability, and film‐forming properties (Muppalla et al., 2014).

Our previous studies with 1.5% CMC showed that it had excellent film‐forming properties; however, the formed films had weak mechanical properties (Fattahi & Seyedain–Ardabili, 2021; Fattahi et al., 2020). The mechanical properties of CMC films can be improved by blending with other polymers. PVA is a versatile polymer with many industrial applications, and it may be the only synthesized polymer whose backbone is mainly composed of C–C bonds that is absolutely biodegradable. Although being a synthetic polymer, PVA has aroused great interest due to its good mechanical properties. So, in order to improve some of the CMC defects and achieve the desired properties, the mentioned polymers are combined to form composite films (Muppalla et al., 2014). However, unlike some other biopolymers such as chitosan, this composite does not have any antimicrobial activities (Raeisi et al., 2015). Yet, this type of packaging can be used as a carrier of active compounds to enhance the safety of foodstuff (Bahrami et al., 2019). Active packaging increases the shelf life of the food without affecting its freshness characteristics. Over the past few years, the use of essential oils as a natural antimicrobial agent in active packaging has received much attention from scientists (Jahed et al., 2017; Raeisi et al., 2015).

Several studies have suggested that incorporation of essential oils into the composite structure may not only yield antimicrobial activity in the film, but they can also increase water vapor resistance (Jahed et al., 2017; Otoni et al., 2014). The first point in the selection of essential oils is their antimicrobial activities. Additionally, aromatic compounds in essential oils are another important point to be considered as these compounds may affect the organoleptic features (Konuk Takma & Korel, 2019).

*Glycyrrhiza glabra* L. originates from the Iranian plateau and other warm regions of the world. Concerning the constituent compounds, the roots of this plant possess a wide range of triterpenes, flavones, isoflavones, saponins, chalcones, glycoside compounds, etc (Siracusa et al., 2011). The unique sweet taste of *Glycyrrhiza* roots is due to glycyrrhizin, which is significantly sweeter than sugar. Nowadays, the roots of *G. glabra* are extensively applied in foods and pharmaceutical industry because of its sweetening, strong antimicrobial, antioxidant, anti‐allergic effects, and other effects (Fatima Khattak & James Simpson, 2010).

However, to the best of our knowledge, the effect of *G. glabra* L. essential oil (GGEO) on the properties of such film‐forming has not been investigated so far. Given the clear importance of the use of natural compounds as an essential oil with strong antimicrobial activity in food packaging, the first objective of this study is to produce a new active composite with the mentioned biodegradable polymer and GGEO. Thereafter, the second goal is to improve the rheological, mechanical, and antimicrobial characteristics of the novel composite by adding a different concentration of essential oils for their potential application as active food packaging.

## 2 EXPERIMENTAL

### 2.1 Materials and bacterial strains

Carboxymethyl cellulose (molecular weight of around 90,000) and PVA (molecular weight range of 89,000–98,000, and 99% hydrolyzed grade) were purchased from Sigma‐Aldrich Company. Glycerol and the salts used to condition the specimens (calcium sulfate, sodium chloride, and magnesium nitrate) were purchased from the Merck. Tween 80 was prepared from Tetrachem Co. GGEO was supplied by Exire Gole Sorkh Pharmaceutical Co. The main volatile compounds of the GGEO were thymol (27.2%), carvacrol (11.1%), eugenol, piperitone (9.4%), p–Vinyl–guaiacol (8.5%), and (4*E*)–decenal (5.3%).

Four pathogenic bacteria, two gram‐positive, *S. aureus* PTCC 1,114 and *Listeria monocytogenes* PTCC 1,163, and two gram‐negative, *E. coli* PTCC 1533 and *Salmonella typhimurium* ATCC 14,028, were selected for the present study. The pure cultures of these bacteria were obtained from the Persian‐type culture collection, Iran. They were all subcultured on tryptic soy broth media (Sigma‐Aldrich).

### 2.2 Preparation of composite films

The solution‐casting method was adopted to prepare binary films. Emulsion mixtures contained 0.25, 0.50, or 0.75% GGEO (dry basis, db), and the ratios of biopolymers–glycerol (plasticizer) and GGEO‐Tween 80 (Emulsifier) were kept constant at 0.75% w/v and 50% v/v respectively during the course of the study (Dashipour et al., 2015). The range of GGEO used was based on its effect on gram‐positive and gram‐negative bacteria in the previous study (Luís et al., 2019).

Each emulsion solution was prepared individually in deionized water. Accordingly, at first, CMC was diluted in distilled water at 1.5% (w/v) with continuous magnetic stirring for 50 min. Further, the PVA solution was prepared by dissolving 5% (w/v) polymer in distilled water under mechanical stirring at 90°C for 120 min. Once the PVA solution was cooled down to the room temperature of 25°C, aqueous solutions were mixed at 50:50 mass ratio under vigorous stirring for 30 min at room temperature of 25°C.

Then, Tween 80 (50% v/v) was added to the composite solutions and mixed at 20,000 rpm for 1 min using an ultra‐homogenizer (Ultraturrax, Janke & Kunkel). Later on, the GGEO was added gradually into the solutions and sonicated through ultrasound probe for 5 min with a working power of 100 W and cycle of 70%. Duty cycle controls the length of each pulse when the sonicator is not in continuous mode. A typical setting is 70%, which means on for 0.7 s, then off for 0.7 s, repeated. Prior to film formation, in order to remove air bubbles from the emulsion solution, a vacuum was used for 10 min. After this step, 80 ml of the resulting emulsion solution was cast into Teflon plates of 10 cm diameter, which was allowed to dry using air convection oven at 40°C for 24 hr. Subsequently, the composites were slowly peeled off from the plates. Once the samples were fabricated, to achieve a relative humidity of 53%, they were conditioned by a saturated solution of magnesium nitrate in a desiccator.

### 2.3 Characterization of the films

#### 2.3.1 Morphological analysis

The morphology and microstructural analysis of the film samples was examined by scanning electron microscopy (*SEM*). Prior to examining the cross section, the filmstrips were cryofractured under liquid nitrogen. Thereafter, the samples were coated with a layer of gold and observed by a scanning electron microscope (MIRA3 ‐ FEG Tescan) operating at an acceleration beam voltage of 100 kV (Bahrami et al., 2019). The cross‐sectional viewpoints of images were captured at slope angles of 90° in relation to the electron beam. Digital micrographs of the surface of composites and cross section were taken at different magnifications whereby the best visualization was chosen (Nisar et al., 2018).

Atomic force microscopy (AFM) experiments were used for topography characterization of composite films previously conditioned by Mg (NO_3_)_2_. In doing so, the Nanosurf, Switzerland atomic force microscope was used. The roughness was estimated using the data from the 3‐D images by Nanosurf Mobile S software.

#### 2.3.2 Fourier transform infrared (FT‐IR) spectroscopy

Fourier transform infrared spectra of all composite samples without and with the addition of GGEO were captured using FT‐IR spectroscopy (TENSOR 27 spectrophotometer and OPUS data collection program, USA). For this purpose, the specimens were cut in 2 cm × 2 cm and then exposed to radiation. The corresponding spectra were assessed within a wavelength range of 4,000–400 cm ^−1^.

#### 2.3.3 Thickness

The thickness of the composite films was determined by a hand‐held digital micrometer (Guanglu Instruments Co.), with a precision of 0.01 mm. The values were taken at 10 random positions for each film. The averages were used to determine the water vapor permeability and mechanical properties.

#### 2.3.4 Dynamic mechanical thermal analysis (DMTA)

Dynamic mechanical thermal analysis analysis was conducted by a dynamic mechanical thermal analyzer (Triton Technology). The test was operated at 1 Hz constant frequency and a strain lower than 0.1 within the temperature range of −100–100°C with a heating rate of 5°C/min. Before the analysis, all films were preconditioned at 53% relative humidity with Mg (NO_3_)_2_ for 24 hr. The storage modulus (E') and loss factor (tan δ) of composite films were obtained as a function of temperature.

#### 2.3.5 Mechanical properties

The tensile properties of the composite film were measured using a tensile tester (Zwick/Roell model FR010) according to the ASTM standard method D882‐97 (Song et al., 2018). The specimens were cut in a dumbbell shape (80 × 5 mm). Prior to testing, samples were conditioned in a desiccator containing saturated Mg (NO_3_)_2_ solution. The separation of the primary knob and crosshead speed was adjusted to 50 mm and 5 mm/min, respectively. Finally, ultimate tensile strength (MPa) and strain at break (%) were measured for each sample. The experiment was performed in three replicates and the mean was reported as the result.

#### 2.3.6 Moisture content and water vapor permeability (MC and WVP)

To measure the moisture content (MC), the samples that were cut into squares of 3 × 3 cm were placed in Petri dishes. Then, the samples were dried in an oven at 105°C for 24 hr. The average of three replicates of different weights of samples was reported as the percentage of moisture content.

A gravimetric method, which was a modified form of ASTM E96–00, was used to determine the water vapor permeability (WVP) of composite films (Otoni, Moura, et al., 2014).

Small cups containing 3 g anhydrous calcium sulfate (0% RH) were sealed by each specimen film (1.7 × 10^−6^ m^2^ exposed film area). The cups were placed in a desiccator containing a saturated salt solution (75% RH). Note that the difference in RH (75% RH) between sodium chloride and calcium sulfate acts as a motive force for water vapor. The cups were then weighed individually every 24 hr. This process lasted for 1 week until the steady state was reached. The weight change of the cups was recorded as a function of time. Water vapor transmission rate (WVTR) was calculated as the slope (g/s) divided by the transfer‐exposed film area (m^2^). The WVP was measured according to the following equation (Nisar et al., 2018):

(1) $$\text{WVP}=\frac{\text{WVTR}}{P\left(R_{1}{\;\text{- R}}_{2}\right)}X$$

where P is the saturation vapor pressure of water (Pa) at the test temperature (25°C), R_1_ is the RH in the desiccator, R_2_ is the RH in the cup, and X is the film thickness (m).

#### 2.3.7 Optical

The color value of composite specimens including lightness (L), redness (a), and yellowness (b) was assessed using a Hunter lab (CR‐300 Minolta Chroma Meter, Minolta Camera Co.). A standard white plate was applied as a reference color during colorimeter calibration (L* = 63, a* = −18 and b* = 2). Hunter value was measured in triplicates, and their average was expressed as the color parameter. Total color difference (ΔE) and whiteness index (WI) were calculated by the following equations (Shojaee‐Aliabadi et al., 2014):

(2) $$\Delta E=\sqrt{{\left(L^{*}‐L\right)}^{2}+{\left(a^{*}‐a\right)}^{2}+{\left(b^{*}‐b\right)}^{2}}$$

(3) $$W1=100‐\sqrt{{\left(100‐L\right)}^{2}}+a^{2}+b^{2}$$

#### 2.3.8 Antibacterial activity

The antimicrobial properties of the samples were evaluated by disk diffusion method. In this method, the inhibition zone around the disk on solid media was used to determine the antimicrobial activity of the film samples against typical selected bacteria. For this purpose, the mentioned bacteria were smeared on Mueller Hinton agar medium at a 10^5^ CFU/ml concentration. In the next step, the specimen films were punched in the form of a disk, 6 mm in diameter, and placed on the seeded solid agar surface. The plates were then incubated at 37°C for overnight.

### 2.4 Statistical analysis

The present study was performed based on a completely randomized design (CRD). Experiments on all treatments were measured in three replications and were expressed as means and standard deviations (mean ± *SD*). Data analysis was performed with the one‐way analysis of variance (ANOVA) using SPSS software version 18.0 (SPSS Inc). Following this step, Duncan's multiple range tests were applied to check the significant difference between treatments at the 95% confidence level.

## 3 RESULTS AND DISCUSSION

### 3.1 *SEM* and AFM

**FIGURE 1:**
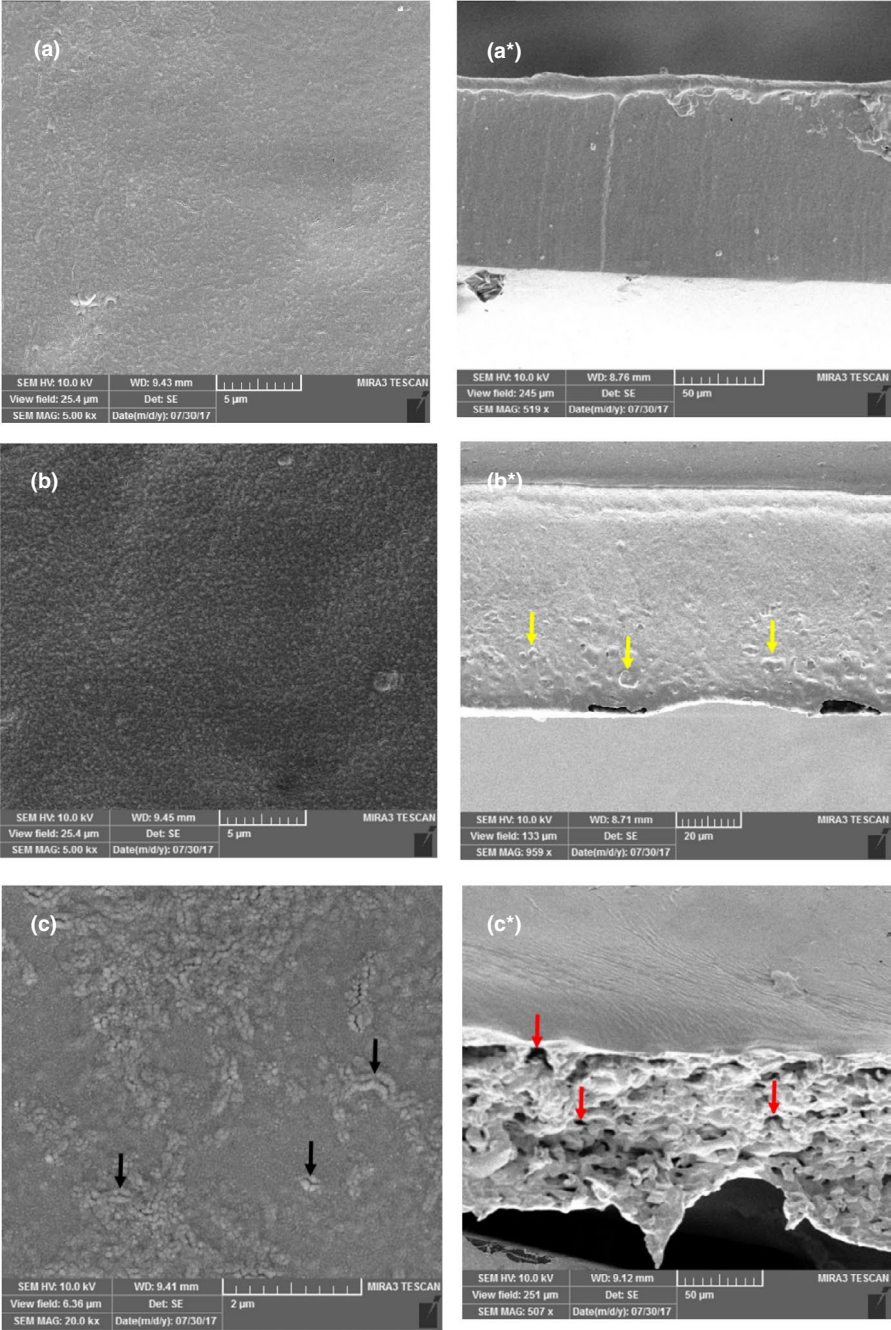
Scanning electron microscopy micrographs of film's surface: (a) control; (b) 0.25% GGEO; (c) 0.75% GGEO and *SEM* micrographs of film's cross sections: (a*) control; (b*) 0.25% GGEO; (c*) 0.75% GGEO. Aggregated droplets (marked with yellow arrows) and pores (marked with red arrows)

The microstructures of the surfaces and cross sections of blend films incorporated with GGEO at different concentrations are displayed in Figure 1. The control film has had a relatively homogeneous and smooth surface without fissures, which is expected for a continuous and cohesive composite (Figure 1a). However, incorporation of GGEO reduced the surface homogeneity of the composite and caused heterogeneity on the surface of the blend films (Figure 1b). The heterogeneity of surface microstructure of the blend films further increased by raising the GGEO content to 0.75% (Figure 1c).

With respect to the cross section, CMC‐PVA composite showed a continuous and compact texture (Figure 1a ^*^), while addition of GGEO into blend films caused a discontinuous and loose texture, with aggregated droplets (marked with yellow arrows) and pores (marked with red arrows) distributed throughout the composite matrix (Figure 1b ^*^,c^*^). On the other hand, when adding a lower amount of the GGEO (Figure 1b ^*^), the film showed an inhomogeneous structure associated with the formation of some independent hydrophobic aggregations (yellow arrows). Meanwhile, the incorporation of the GGEO at a higher concentration (Figure 1c ^*^) caused a sponge‐like structure, with micropores (red arrows) distributed throughout the film matrix. These structural discontinuities could be related to the formation of the two phases in the film matrix, due to the hydrophobic nature of the EO. It seems that at higher amounts of the GGEO, the oil droplets caused clumps as a result of higher collision frequency among them, subsequently increasing the creaming rates. According to the Stokes' law, the migration rate of the droplets is proportional to the square of their radius (Homayoonfal et al., 2015). Hence, by increasing the droplet size, the creaming rate of the droplets increased. Occurrence of phase separation (EO droplets and composite) and creaming of EO droplets occurred in the film matrix, driving part of the droplets toward the blend film's surface (marked with black arrows), (Figure 1c) where essential oil evaporation occurs together with water molecules (Lee et al., 2018). Nisar et al., (2018) have reported similar results when clove bud essential oil was incorporated into citrus pectin films.

**FIGURE 2:**
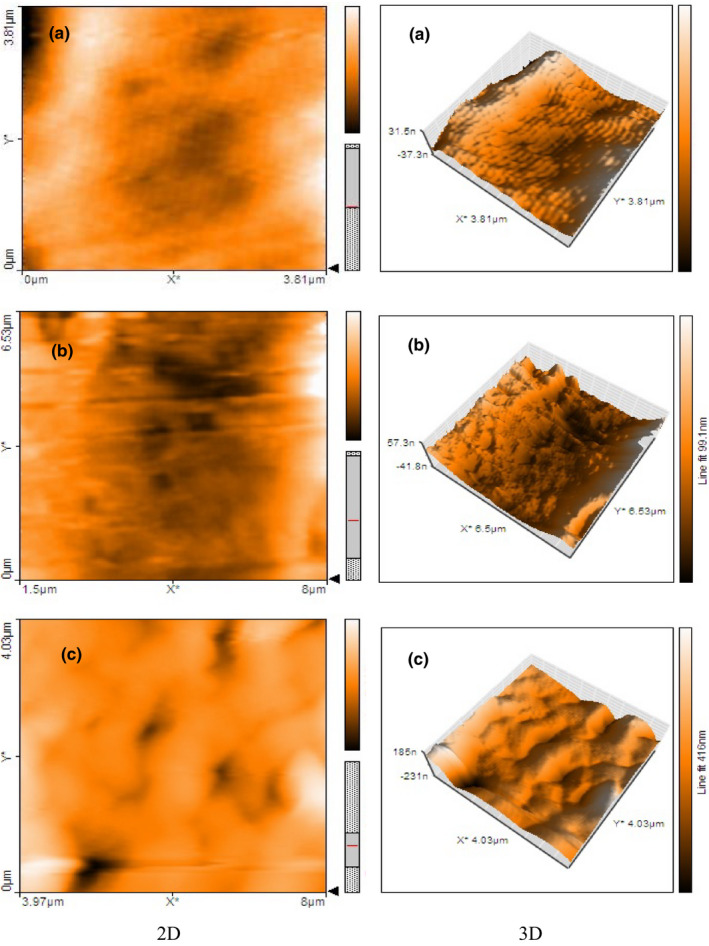
Atomic force microscopy (2‐D and 3‐D) plots of surface morphologies of control film (a) and blend films with 0.25% GGEO (b) and 0.75% GGEO (c)

Atomic force microscopy is further used to clarify the descriptive information obtained by *SEM*. The calculations of roughness indices can be used to provide quantitative information regarding the surface morphology of composite at the nanometer scale (Jahed et al., 2017). The roughness of the films is often critical to their applications, and in particular, roughness strongly impacts their optical, barrier, and frictional properties. Table 1 reports the results of roughness indices, the average (R_a_) plus root–mean–square (R_q_) roughness for blend films with and without essential oil. In addition, the corresponding plots (2‐D and 3‐D) of surface morphologies are depicted in Figure 2.

**TABLE 1:** Roughness indices obtained from Nanosurf Mobile S software for blend films with and without GGEO

Sample name	Roughness indices (nm)	
	R_a_	R_q_
CMC‐PVA	7.60 ± 3.12^b^	8.87 ± 3.83^b^
CMC‐PVA−0.25% GGEO	11.79 ± 4.60^b^	14.07 ± 5.14^b^
CMC‐PVA−0.75% GGEO	27.04 ± 9.21^a^	36.57 ± 16.15a

Note

Data are given as means and standard deviation. Different superscript letters indicate the significant difference of values in the same column (*p* < .05).

According to Table 1 along with the 2‐D and 3‐D plots (Figure 2a), blend films without GGEO had a relatively smooth surface. The R_a_ of the control film was 7.60 nm and increased significantly (*p* < .05) to 27.04 nm for blend films containing 0.75% GGEO. Likewise, the R_q_ was enhanced from 8.87 nm to 36.57 nm. These results could be due to the migration of aggregated essential oil droplets to the blend film's surface during the drying process (Figure 2b,c), which was in accordance with *SEM* analysis. Some studies have also obtained similar results for kappa carrageenan with *Satureja hortensis* EO, poly (lactic acid) with *Origanum vulgare L* EO, and gelatin with olive oil (Javidi et al., 2016; Ma et al., 2012; Shojaee‐Aliabadi et al., 2014).

### 3.2 FT‐IR

**FIGURE 3:**
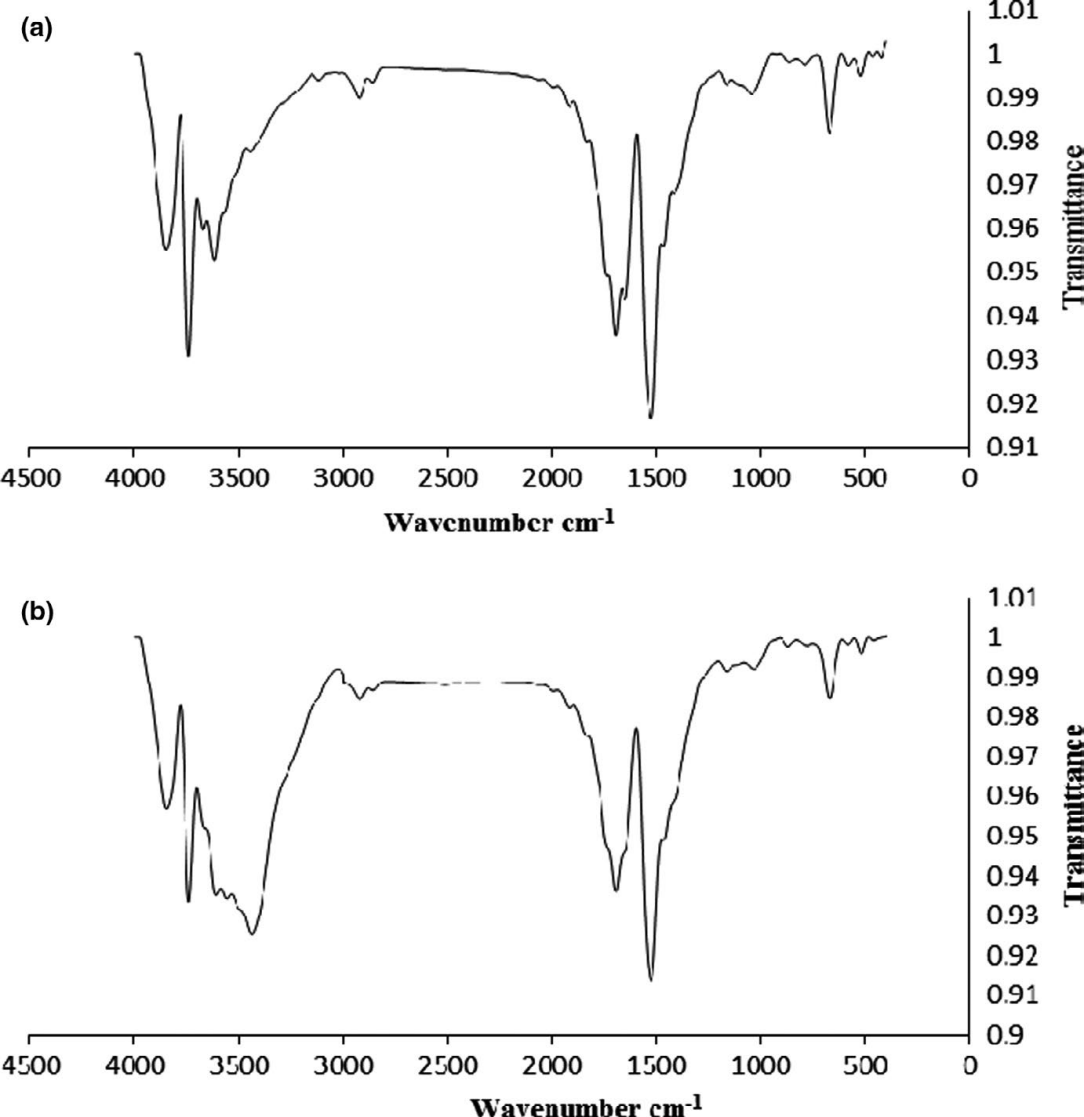
Fourier transform infrared spectra of control film (a) incorporated with 0.75% GGEO (b)

The FT‐IR spectra of films were used to study the structure of composites and confirm the interaction between the matrix of polymers and GGEO over the wavenumber range 4,000–400 cm^‐1^. The spectra of neat film and composites containing different concentrations of GGEO are displayed in Figure 3. Both CMC and PVA possess some common functional groups in their structure; therefore, the group areas of FT‐IR spectra are in relatively similar locations and their spectra vary generally in the fingerprint regions. As can be seen from the figure, the peaks located within the wavenumber 3,500–3,800 cm^‐1^ region were found in both samples, related to the stretching vibration of wide‐ranging hydroxyl groups (‐OH) of polymers and GGEO. The sharp peak at 1,500 cm^‐1^ can be attributed to symmetrical stretch of acidic groups (COO‐) in GGEO and the combination of polymers. Further, the strong peaks observed around region 1,600 cm^‐1^ might be associated with the C‐O of the six‐carbon cyclic pyranose of CMC and GGEO. In the sample containing essential oil, the peaks are also integrated into the region within 1,600–1,000 cm^‐1^. This indicates the interaction between the functional groups of polymers and functional groups of GGEO. Therefore, as shown in the figure in this area, the peak corresponding to these functional groups has been considerably broader.

### 3.3 Thickness

According to the literature, the thickness of the films is affected by the compatibility between polymer and filler, the number of solid contents, and free volumes inside the film matrix (Chu et al., 2019; Nisar et al., 2018). According to Table 2, the thickness of the control film was equal to 0.148 mm. The thickness slightly increased by addition of 0.25% and 0.50% GGEO into the CMC‐PVA composite. According to the results, this change was not significant compared with the control film (*p* > .05). However, with elevation of the GGEO content to 0.75%, the thickness was significantly enhanced up to 0.201 mm (*p* < .05). It seems that the addition of GGEO into the composite matrix might reduce the interaction between polymer chains. This is probably due to the aggregation and creaming of GGEO droplets, which contribute to the formation of many free spaces in the composite matrix. This causes formation of an uncompact and sponge‐like structure thereby increasing the thickness of the blend films. These results can be confirmed by the cross‐sectional images of *SEM* (Figure 1b ^*^,c^*^). These findings were consistent with the results of adding clove and cinnamon essential oil into the agar and gelatin‐based films, respectively (da Rocha et al., 2018; Wu et al., 2017). They reported that the increase in film thickness could be due to the generation of incompatibilities between the polymer and essential oil, leading to a less dense structure and thus increased film thickness.

**TABLE 2:** Thickness, mechanical properties, moisture content (MC), and water vapor permeability (WVP) of the control film and blend films with GGEO

Sample name	Thickness (mm)	Mechanical properties		MC (%)	WVP ×10^–9^ (g/m s^−1^ Pa^−1^)
		SAB (%)	UTS (MPa)		
CMC‐PVA	0.148 ± 0.04^b^	120.87 ± 19.19^c^	17.01 ± 1.21^a^	32.12 ± 1.01^b^	7.25 ± 1.01^b^
CMC‐PVA−0.25% GGEO	0.164 ± 0.02^ab^	168.28 ± 14.40^b^	13.08 ± 3.58^b^	36.64 ± 1.02^ab^	12.68 ± 1.06^a^
CMC‐PVA−0.50% GGEO	0.182 ± 0.02^ab^	215.69 ± 17.27^a^	8.47 ± 1.72^c^	37.70 ± 5.59^ab^	11.82 ± 2.25^a^
CMC‐PVA−0.75% GGEO	0.201 ± 0.05^a^	236.12 ± 26.04^a^	3.86 ± 1.01^d^	41.06 ± 1.21^a^	13.61 ± 1.78^a^

Note

Data are given as means and standard deviation. Different superscript letters indicate the significant difference of values in the same column (*p* < .05).

### 3.4 DMTA

**FIGURE 4:**
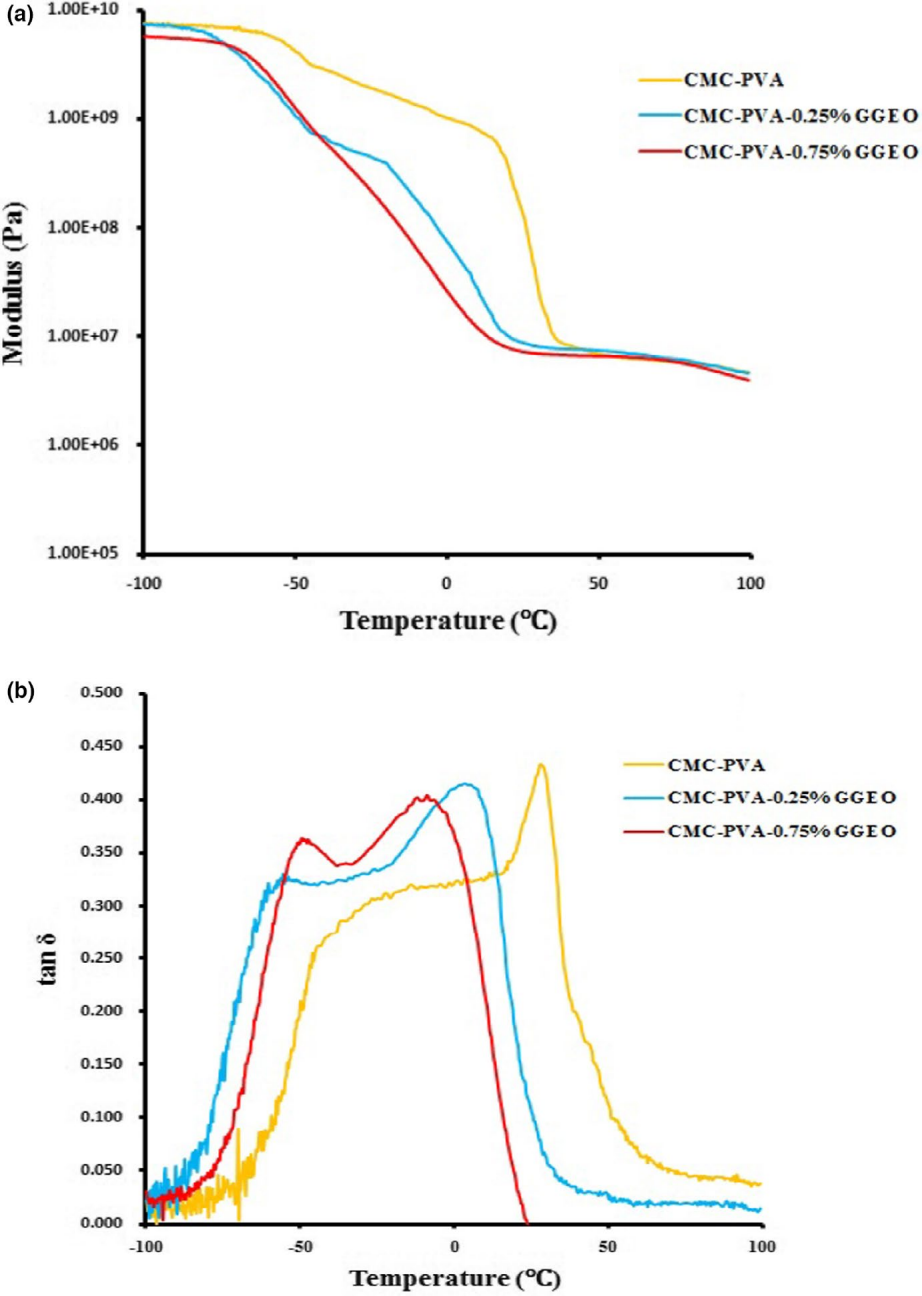
Storage modulus (a) and tan δ (b) versus temperature curves of CMC‐PVA and CMC‐PVA‐GGEO blend films

Figure 4a demonstrates the storage modulus versus temperature curves of the blend films containing 0.25% and 0.75% GGEO, as well as the control film. The storage modulus is directly related to the stiffness of the final composite (Hasheminya et al., 2019). All the curves show the reduction of storage modulus as the temperature rises until reaching around 40°C. Meanwhile, with a further increase in temperatures, the softening of the plasticized composite masked the GGEO plasticizing effect and all curves tended to merge.

Further, the addition of GGEO led to a sharp reduction of the slope of the storage modulus curves in relation to the corresponding neat CMC‐PVA composite. It indicates that EO acts as a plasticizer in the composite matrix and improves the chain mobility (Mosnáčková et al., 2019). As a result, the addition of GGEO to the composite matrix reduced blend film's stiffness. Similar results have been shown for sodium caseinate films plasticized with linseed oil resin (Pereda et al., 2015).

The curves for tan δ as a function of temperature for the composites containing 0.25% and 0.75% GGEO, as well as the control film, are indicated in Figure 4b. Solid‐to‐liquid state transition temperature can be calculated from the maximum value of tan δ (loss factor) curve (Sahraeian et al., 2019). The higher‐temperature peak of loss factor has corresponded to the glass transition temperature (T_g_) of CMC‐PVA composite. T_g_ is related to the segmental mobility of the molecular chains in amorphous regions. (Salarbashi et al., 2018). In a study, as the GGEO concentration increased, the glass transition temperature decreased, and also compared to the neat CMC‐PVA composite the loss peaks tended to broaden. Due to the migration of GGEO droplets, many empty voids were generated in the matrix of the composite. As mentioned earlier, by increasing the number of free volumes, the chain mobility of the blend films was enhanced (Bahrami et al., 2019). These results were consistent with the findings of Zinoviadou et al., (2009) who reported a similar behavior due to the plasticizing action of oregano oil on the thermo‐mechanical properties of whey protein isolate films.

### 3.5 Mechanical properties

The study of mechanical properties such as ultimate tensile strength (UTS) and strain at break (SAB) can help predict the stress tolerance and structural integrity of composites (da Rocha et al., 2018). The effect of GGEO added at different concentrations on the mechanical properties of the blend films is summarized in Table 2. As shown from the results, the addition of GGEO improved the flexibility of blend films, which increased by about twice compared to the control film. As can be observed, when GGEO density reached 0.75%, the SAB significantly increased (*p* < .05) from 120.87% to 236.12%. On the contrary, the UTS significantly diminished from 17.01 MPa to 3.86 MPa (*p* < .05).

The changes observed in SAB and UTS in the blend films are related to inter‐ and intra‐molecular forces as well as the network microstructure (Abdollahi et al., 2019; Han et al., 2018). The incorporation of GGEO into the CMC‐PVA matrix increases the free volume and chain mobility of the polymers. These results were in agreement with the phenomenon discussed earlier for DMTA analysis. In conclusion, EO due to its lipid nature acts as a plasticizer in the composite matrix and can be easily deformed in the blend film structure (Song et al., 2018).

Based on *SEM* data, when adding a lower amount of 0.25% of GGEO, aggregation was observed in the blend film matrix (Figure 1b ^*^). In a study, increasing the content of GGEO caused cracking on the site of evaporation of the oil on the surface of the film (Figure 1c). These irregularities can most likely weaken the composite structure and reduce the mechanical strength of the blend films (Alizadeh‐Sani et al., 2018). The review of the literature suggested that the results of several studies are consistent with ours (Song et al., 2018; Wu et al., 2017).

### 3.6 MC and WVP

Characteristics such as moisture content (MC) and water vapor permeability (WVP) are important physical properties to assess water retention by the composite matrix and the water diffusion through the composite, respectively (Li et al., 2018). Generally, the lower the values of these parameters, the higher the efficiency of the film is as a moisture barrier (Bastos et al., 2016). The MC and WVP values of the control and GGEO incorporated blend films are reported in Table 2. The MC of the neat CMC‐PVA composite was 32.12% showing an insignificant difference with the blend films incorporated with 0.25%–0.50% of essential oil (*p* > .05). However, MC was significantly increased up to 41.06% (*p* < .05) after incorporation of 0.75% GGEO. These results are in agreement with the data reported by Jouki et al., (2014). According to them, these results were attributed to the breakdown of the film structure by the presence of EO. In this way, more water molecules are confined among polymer chains via hydrogen bonding.

Likewise, the WVP values of the blend films increased by incorporation of GGEO in the composite matrix (Table 2). The WVP of the CMC‐PVA films significantly increased (*p* < .05) from 7.25 × 10^−9^ g/m s^−1^ Pa^−1^ to 12.68 × 10^−9^ g/m s^−1^ Pa^−1^ as the concentration of GGEO rose from 0% to 0.25%. In addition, the WVP of the blend films increased slightly (*p* > .05) from 12.68 × 10^−9^ g/m s^−1^ Pa^−1^ to 13.61 × 10^−9^ g/m s^−1^ Pa^−1^ at GGEO contents over 0.25%. According to the *SEM* images (Figure 1), this result can be explained by the discontinuities occurring in the composite structure by improper dispersion of essential oil droplets. This could increase the amount of water vapor transfer, resulting in the WVP increase (Hosseini et al., 2015).

A similar result has been reported by Dashipour et al., (2015) where the effect of *Zataria multiflora* essential oil on WVP of CMC film was observed. In addition, investigating the effect of summer savory essential oil of WVP of CMC‐agar biocomposite films, Abdollahi et al., (2019) achieved similar results. On the other hand, this may be due to the lubricating effect of EO and thus increasing the free spaces in the composite network, allowing the passage of water vapor molecules. This is similar to the finding described by other researchers and has been verified via mechanical and DMTA results (Ghadetaj et al., 2018; Lee et al., 2019).

### 3.7 Optical

Colorimetric parameters of the blend films, including Hunter Lab color values (L, a, b), the total difference in color (ΔE), whiteness index values (WI), and yellowness index values (YI), are provided in Table 3. As shown, the addition of essential oil into the composite matrix led to a marked drop in both the WI and Hunter L‐values of the blend films. However, YI, Hunter a‐, and b‐values significantly rose with an increase in essential oil. Therefore, the incorporation of GGEO increased the darkness, yellowness, and redness of blend films as compared with the control film. These optical changes can be related to the yellowish and reddish color of GGEO, absorbing light at a lower wavelength. This property could help protect the packaged foods from visible light and ultraviolet rays, thus reducing the photo‐oxidation reactions, discoloration, and nutrient loss (Zhang et al., 2018).

**TABLE 3:** Effect of different amounts of GGEO on color parameters of blend films

Sample name	Hunter lab color values			ΔE	WI	YI
	L	a	B			
CMC‐PVA	55.99 ± 1.01^a^	−13 ± 0.01^d^	1.99 ± 1.01^d^	14.69 ± 2.14^d^	54.10 ± 1.01^a^	2.55 ± 1.01^d^
CMC‐PVA−0.25% GGEO	48.33 ± 2.51^b^	−11 ± 0.01^c^	13.33 ± 2.51^c^	25.67 ± 3.71^c^	45.58 ± 3.13^b^	39.73 ± 9.48^c^
CMC‐PVA−0.50% GGEO	47.16 ± 1.01^b^	−6 ± 0.50^b^	23.67 ± 1.52^b^	34.28 ± 2.56^b^	40.87 ± 1.31^c^	72.66 ± 4.45^b^
CMC‐PVA−0.75% GGEO	45.99 ± 1.01^b^	1 ± 0.99^a^	34.01 ± 1.01^a^	42.89 ± 2.16^a^	36.16 ± 1.01^d^	105.60 ± 2.77^a^

Note

Data are given as means and standard deviation. Different superscript letters indicate the significant difference of values in the same column (*p* < .05).

In some studies, a similar trend has been reported with the addition of essential oils to packaging films, for example chitosan with turmeric essential oil (Li et al., 2019), soy protein with clove essential oil (Ortiz et al., 2018), and chitosan with *Perilla frutescens* (L.) Britt. essential oil (Zhang et al., 2018). Generally, the incorporation of GGEO at different concentrations in a composite matrix intensified the color parameters, such that the ΔE of blend films significantly increased. Such a behavior has also been reported in previous studies (Jahed et al., 2017; Shojaee‐Aliabadi et al., 2014).

### 3.8 Antibacterial activity

**FIGURE 5:**
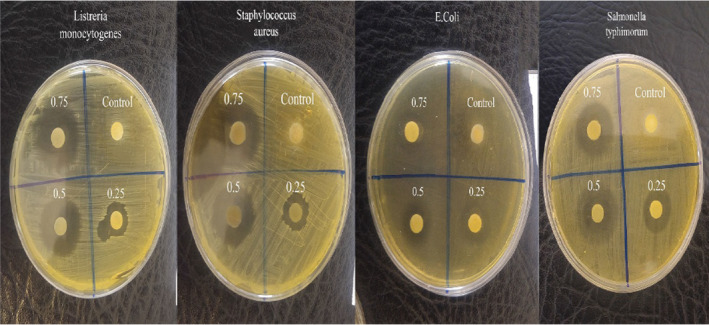
Antimicrobial activity of composite films loaded with different percentage of GGEO against selected gram‐positive and gram‐negative bacteria

The antimicrobial activity of the fabricated films was measured using a disk diffusion method and measuring the inhibition zone around each sample in media disk. Figure 5 illustrates the antibacterial properties of CMC‐PVA composites with and without various concentrations of GGEO against selected gram‐positive (*L. monocytogenes* and *S*. *aureus*) and gram‐negative (*E. coli* and *S. typhimurium*) bacteria. As can be seen, the neat blend film lacks antibacterial activity. In addition, inhibition zones increased significantly, with gradual elevation of GGEO concentration. This clearly demonstrated that the ability of the active compounds in the essential oil to inhibit the gram‐positive and gram‐negative bacteria depends on its concentration in composites (Ahmad et al., 2012). At high concentrations (0.75%), the greater penetration of the active compounds of the GGEO into the media prevents the growth of bacteria whereby the inhibition zone has grown larger. The antimicrobial activity of GGEO was due to a wide range of phenolic compounds such as flavonoids and isoflavones (Fatima Khattak & James Simpson, 2010). Several studies have revealed that the antimicrobial activity of essential oils is probably due to an attack on phospholipid in cell membranes (Ahmad et al., 2012; Jahed et al., 2017; Ma et al., 2015). Consequently, the permeability of the membrane increases and the contents of the cell's cytoplasm are dripped. On the other hand, there is a possibility of their interaction with enzymes in the cell wall thereby inactivating them (Ahmad et al., 2012).

Films with GGEO, especially at higher concentrations, were more effective against all tested bacteria than the control film. Since the films obtained from CMC and PVA do not have antimicrobial activity, it can be concluded that the addition of GGEO at different concentrations into the composites causes the production of appropriate antimicrobial properties in them.

## 4 CONCLUSIONS

In present study, the fabrication and characterization of CMC‐PVA‐based composite films for food antimicrobial packaging application were carried out. Generally, chemical structure and morphological properties of CMC‐PVA‐based films were affected by GGEO (as active agent), which was confirmed by FT‐IR, *SEM*, and AFM testing. Furthermore, ultimate tensile strength, storage modulus, and glass transition temperature of CMC‐PVA‐based films were decreased by addition of EO and these films showed a tendency to yellowing, with a significant increase in darkness and less WI than the control film. Microbial tests represented superior antibacterial activity of composite films especially at the highest concentrations of GGEO (0.75%). Nevertheless, further studies are required to improve the hydrophobicity and moisture barrier of derived films.

## CONFLICT OF INTEREST

There is no conflict of interest to declare.
